# Stem cell therapies for neurological disorders: current progress, challenges, and future perspectives

**DOI:** 10.1186/s40001-024-01987-1

**Published:** 2024-07-25

**Authors:** Ramyar Rahimi Darehbagh, Seyedeh Asrin Seyedoshohadaei, Rojin Ramezani, Nima Rezaei

**Affiliations:** 1 Student Research Committee, Kurdistan University of Medical Sciences, Sanandaj, Iran; 2 Nanoclub Elites Association, Tehran, Iran; 3 Cellular and Molecular Research Center, Research Institute for Health Development, Kurdistan University of Medical Sciences, Sanandaj, Iran; 4 Universal Scientific Education and Research Network (USERN), Sanandaj, Kurdistan Iran; 5 Kurdistan University of Medical Sciences, Sanandaj, Iran; 6 Network of Immunity in Infection, Malignancy and Autoimmunity (NIIMA), Universal Scientific Education and Research Network (USERN), Tehran, Iran; 7 Research Center for Immunodeficiencies, Children’s Medical Center, Tehran University of Medical Sciences, Dr. Qarib St, Keshavarz Blvd, Tehran, 14194 Iran; 8 Department of Immunology, School of Medicine, Tehran University of Medical Sciences, Tehran, Iran

**Keywords:** Stem cell therapy, Neurological disorders, Paracrine effects, Immunomodulation, Clinical translation

## Abstract

Stem cell-based therapies have emerged as a promising approach for treating various neurological disorders by harnessing the regenerative potential of stem cells to restore damaged neural tissue and circuitry. This comprehensive review provides an in-depth analysis of the current state of stem cell applications in primary neurological conditions, including Parkinson’s disease (PD), Alzheimer’s disease (AD), amyotrophic lateral sclerosis (ALS), multiple sclerosis (MS), stroke, spinal cord injury (SCI), and other related disorders. The review begins with a detailed introduction to stem cell biology, discussing the types, sources, and mechanisms of action of stem cells in neurological therapies. It then critically examines the preclinical evidence from animal models and early human trials investigating the safety, feasibility, and efficacy of different stem cell types, such as embryonic stem cells (ESCs), mesenchymal stem cells (MSCs), neural stem cells (NSCs), and induced pluripotent stem cells (iPSCs). While ESCs have been studied extensively in preclinical models, clinical trials have primarily focused on adult stem cells such as MSCs and NSCs, as well as iPSCs and their derivatives. We critically assess the current state of research for each cell type, highlighting their potential applications and limitations in different neurological conditions. The review synthesizes key findings from recent, high-quality studies for each neurological condition, discussing cell manufacturing, delivery methods, and therapeutic outcomes. While the potential of stem cells to replace lost neurons and directly reconstruct neural circuits is highlighted, the review emphasizes the critical role of paracrine and immunomodulatory mechanisms in mediating the therapeutic effects of stem cells in most neurological disorders. The article also explores the challenges and limitations associated with translating stem cell therapies into clinical practice, including issues related to cell sourcing, scalability, safety, and regulatory considerations. Furthermore, it discusses future directions and opportunities for advancing stem cell-based treatments, such as gene editing, biomaterials, personalized iPSC-derived therapies, and novel delivery strategies. The review concludes by emphasizing the transformative potential of stem cell therapies in revolutionizing the treatment of neurological disorders while acknowledging the need for rigorous clinical trials, standardized protocols, and multidisciplinary collaboration to realize their full therapeutic promise.

## Introduction

Neurological disorders encompass a wide range of debilitating conditions that affect the central and peripheral nervous systems, leading to progressive damage and loss of neural tissue. These conditions include neurodegenerative illnesses, which are typified by the build-up of abnormal protein aggregates and the progressive loss of particular neuronal populations. Examples of these illnesses are Alzheimer’s disease (AD), Parkinson’s disease (PD), and Huntington’s disease (HD) [1, 2]. Other neurological conditions, such as multiple sclerosis (MS) and spinal cord injury (SCI), involve damage to the myelin sheath and axons, disrupting neural transmission and causing functional impairments [3, 4]. Cerebrovascular disorders, including stroke and traumatic brain injury (TBI), result in acute neural tissue damage and subsequent neuroinflammation, leading to long-term disability [5, 6]. Neurological disorders pose a significant burden on global health, affecting millions of individuals worldwide and leading to substantial healthcare costs and societal impact [7, 8].

Current therapeutic approaches for neurological disorders primarily focus on managing symptoms and slowing disease progression rather than addressing the underlying pathology. This is in part because the precise etiology of many neurological conditions remains unknown, limiting our ability to develop targeted disease-modifying therapies. Pharmacological treatments, such as dopaminergic medications for PD, cholinesterase inhibitors for AD, and immunomodulatory drugs for MS, provide symptomatic relief but often have limited efficacy and side effects [9–11]. Rehabilitation strategies aim to promote functional recovery and adaptations but do not directly restore lost neural tissue [12]. Surgical interventions, such as deep brain stimulation for PD, can alleviate specific symptoms but do not halt or reverse the neurodegenerative process [13]. While progress has been made in developing neuroprotective agents and gene therapies, their clinical translation has been challenging, and their long-term efficacy remains to be established [14, 15]. Given the limitations of current therapies, there is a pressing need for novel approaches that can effectively regenerate damaged neural tissue, replace lost neurons, and promote functional recovery in neurological disorders.

By utilizing stem cells' capacity for regeneration, stem cell-based therapies have become a viable option for treating the underlying pathophysiology of neurological illnesses. Because of their capacity to self-renew and differentiate into distinct cell types, stem cells are desirable in regenerative medicine [16]. Numerous stem cell types have been investigated for their potential as therapeutics for neurological illnesses, including induced pluripotent stem cells (iPSCs), neural stem cells (NSCs), mesenchymal stem cells (MSCs), and embryonic stem cells (ESCs) [17, 18]. These cells offer a variety of alternatives for cell-based therapeutics since they can be produced from various sources, including adult bone marrow, adipose tissue, embryonic tissue, and reprogrammed somatic cells [19, 20]. Stem cells have a variety of therapeutic applications in neurological illnesses, including immunomodulation, cell replacement, paracrine signaling, and stimulation of endogenous repair mechanisms [21–23]. Preclinical studies in animal models have demonstrated the ability of stem cells to differentiate into neuronal and glial lineages, integrate into host neural circuits, and promote functional recovery in various neurological conditions [24, 25]. However, the clinical translation of stem cell therapies faces numerous challenges, including optimizing cell manufacturing, delivery methods, and safety assessments [26].

This comprehensive review aims to provide an in-depth analysis of the current state of stem cell-based therapies for neurological disorders, focusing on the most recent advances and clinical applications. The review will discuss the biological properties and therapeutic mechanisms of different stem cell types, critically examine the preclinical and clinical evidence for their efficacy and safety, and highlight the challenges and future directions in the field. By synthesizing the latest research findings and expert opinions, this review seeks to inform researchers, clinicians, and stakeholders about the potential and limitations of stem cell therapies in revolutionizing the treatment of neurological disorders.

## Stem cell basics

Stem cells are unspecialized cells with the unique ability to self-renew and differentiate into various cell types, making them a valuable tool for regenerative medicine [27]. Understanding stem cells' fundamental properties and mechanisms is crucial for their therapeutic application in neurological disorders. This section provides an overview of the different types of stem cells, their sources, and their mechanisms of action in neurological therapies.

Based on their potential for differentiation and developmental stage, stem cells can be categorized. All of the body's cell types can be produced by ESCs, pluripotent cells formed from the inner cell mass of blastocysts [28]. Nevertheless, there are hazards of tumor growth and ethical issues with using ESCs [29]. Adult stem cells are multipotent cells in bone marrow, adipose tissue, and the central nervous system. Examples of these tissues are MSCs and NSCs [30, 31]. Though less contentious and safer than ESCs, these cells have a more constrained capacity for differentiation [32]. Adult somatic cells are reprogrammed into a pluripotent state using particular transcription factors to create iPSCs [33]. iPSCs possess similar properties to ESCs but avoid the ethical issues associated with embryonic tissue use [34]. Other stem cell sources include perinatal tissues, such as umbilical cord blood and amniotic fluid, which contain a mix of multipotent stem cells [35].

Stem cells exert their therapeutic effects in neurological disorders through multiple mechanisms, broadly categorized into cell replacement, paracrine signaling, immunomodulation, and stimulation of endogenous repair processes.

1. Cell replacement: Stem cells can differentiate into specific neuronal and glial cell types, potentially replacing damaged or lost neural cells in neurological disorders [36]. For instance, dopaminergic neurons derived from stem cells can be transplanted into the striatum to replace degenerated neurons in Parkinson's disease [37]. However, the extent of cell replacement and functional integration of transplanted cells varies across different neurological conditions and requires further optimization [38].
2. Paracrine signaling: Stem cells secrete a wide range of bioactive molecules, including growth factors, cytokines, and extracellular vesicles, which can exert neuroprotective, anti-inflammatory, and regenerative effects on the host neural tissue [39, 40]. These paracrine factors can promote the survival and regeneration of endogenous neural cells, modulate the immune response, and enhance angiogenesis and neuroplasticity [41, 42]. The paracrine mechanisms of stem cells are believed to play a crucial role in their therapeutic efficacy, particularly in conditions where cell replacement alone may not be sufficient [43].
3. Immunomodulation: Neuroinflammation is a common feature of many neurological disorders, contributing to neural damage and hindering repair processes [44]. Stem cells, particularly MSCs, possess immunomodulatory properties that can regulate the immune response and create a more favorable environment for neural repair [45]. These cells can interact with various immune cells, such as T, B, and microglia, and modulate their activity through direct cell–cell contact and secretion of soluble factors [46, 47]. By attenuating neuroinflammation and promoting a pro-regenerative immune response, stem cells can indirectly support neural repair and functional recovery [48].
4. Stimulation of endogenous repair: To encourage the proliferation, differentiation, and integration of endogenous stem and progenitor cells into the injured neural tissue, stem cells can activate and mobilize these cells in the brain [49, 50]. Growth factors and chemokines that draw endogenous stem cells to the injury site and promote their survival and differentiation can be secreted to do this [51]. Furthermore, stem cells can expand the brain's neurogenic and angiogenic niches, improving the conditions for endogenous repair mechanisms [52].

The therapeutic mechanisms of stem cells in neurological disorders are complex and multifaceted, often involving a combination of cell replacement, paracrine signaling, immunomodulation, and stimulation of endogenous repair. The relative contribution of each mechanism may vary depending on the specific neurological condition, the type of stem cells used, and the route and timing of administration [53]. Understanding these mechanisms is crucial for optimizing stem cell-based therapies and developing targeted approaches for neurological disorders.

However cell-based therapy for neurological illnesses encounters various obstacles despite its considerable potential:

- The potential of developing tumours, especially with pluripotent stem cells, is a concern [54]
- Allogeneic cell transplants can be rejected by the immune system [55]
- Cell survival and incorporation in the host tissue are restricted
- The possibility of unregulated differentiation or movement [56]
- There are ethical considerations related to the utilisation of embryonic stem cells [57]
- Issues related to scalability and manufacture of cell products that meet clinical-grade standards [58].

There are questions about the safety of anything over a long period of time, and it is necessary to do additional studies to continue monitoring it [59].

These constraints highlight the significance of thorough preclinical testing and meticulous clinical trial design. Furthermore, it is imperative for regulatory frameworks to adapt in order to effectively tackle the distinctive obstacles presented by cell-based therapies [20].

Each type of stem cell has unique benefits and drawbacks when it comes to its use in neurological applications. ESCs possess a significant degree of adaptability, but they can give rise to ethical dilemmas and pose the possibility of developing tumours [60]. Adult stem cells, such as MSCs and NSCs, have a narrower range of cell types they can develop into, but they may present fewer safety risks [61]. iPSCs offer a means of obtaining cells that are particular to each patient, but they must undergo thorough analysis to guarantee their safety and effectiveness [31]. When selecting a cell type, it is important to thoroughly evaluate the individual neurological disease and therapeutic objectives [35].

## Stem cell therapy in specific neurological diseases

### Alzheimer’s disease

Amyloid-beta (Aβ) plaques and neurofibrillary tangles build up in Alzheimer's disease (AD), a progressive neurodegenerative condition that impairs memory, causes neuronal death and declines cognitive function [62]. Current pharmacological treatments, such as cholinesterase inhibitors and memantine, provide symptomatic relief but do not address the underlying pathology or halt disease progression [63]. Stem cell-based therapies have been proposed as a potential strategy to address multiple aspects of Alzheimer's disease pathology, including replacing lost neurons, providing neuroprotection, and modulating neuroinflammation [64].

#### Preclinical studies

Preclinical studies using animal models of AD have demonstrated the potential of various stem cell types, including MSCs, NSCs, and iPSCs, to ameliorate AD pathology and improve cognitive function. MSCs have been shown to reduce Aβ deposition, attenuate neuroinflammation, and promote neurogenesis and synaptic plasticity in AD mouse models [65–67]. NSCs derived from human fetal tissue or differentiated from pluripotent stem cells have been reported to differentiate into cholinergic neurons and integrate into the host brain, improving cognitive function in AD animal models [68, 69]. However, transplanted cells' long-term survival and functional integration remain challenging [70]. iPSC-derived neural cells have also shown promise in preclinical studies, with the advantage of allowing patient-specific and genetically corrected cell therapies [71, 72].

#### Clinical trials

To date, only a limited number of small-scale clinical trials have investigated the safety and feasibility of stem cell therapies in AD patients. A phase I trial using human umbilical cord blood-derived MSCs (hUCB-MSCs) demonstrated the safety and tolerability of repeated intravenous infusions in AD patients, with some evidence of stabilization of cognitive function [73]. Another phase I trial using autologous adipose-derived MSCs (ADSCs) showed safety and potential efficacy in slowing cognitive decline in mild to moderate AD patients [74]. However, these early-stage trials have limitations, such as small sample sizes, lack of placebo controls, and short follow-up periods, making it difficult to draw definitive conclusions about the efficacy of stem cell therapies in AD [75].

#### Challenges and future directions

Obstacles and prospects for the future although the preclinical results show promise, various hurdles must be overcome to successfully apply stem cell therapy in the clinical treatment of AD. These include enhancing the efficiency and quality of stem cells, enhancing the viability and effective integration of transplanted cells, and devising precise delivery techniques to specific brain regions impacted by AD [76]. Additionally, the optimal timing of intervention, the long-term safety and efficacy, and the potential need for repeated treatments need to be established through well-designed clinical trials [67].

Future directions in stem cell therapy for AD may involve using genetically modified stem cells to enhance their therapeutic properties, such as increased secretion of neurotrophic factors or Aβ-degrading enzymes [77]. Combining stem cell therapy with other therapeutic approaches, such as Aβ immunization or small molecule inhibitors of Aβ and tau pathology, may provide synergistic benefits [78]. 3D organoid models derived from patient-specific iPSCs may also facilitate drug screening and personalized treatment strategies [79].

Although stem cell therapy shows potential as a disease-modifying treatment for AD, additional research is required to tackle the obstacles and enhance the therapeutic strategy. Thorough preclinical investigations and well-planned clinical trials are necessary to determine stem cell treatments' safety, effectiveness, and long-term advantages in Alzheimer's disease. However, the complex and multifaceted nature of AD presents significant challenges for developing effective cell replacement therapies. AD involves widespread neuronal loss, synaptic dysfunction, protein aggregation, and vascular abnormalities across multiple brain regions. Simple cell replacement is unlikely to address all of these pathological features. Additional research is required to determine if stem cell approaches can tackle the numerous obstacles presented by AD's complexity and enhance therapeutic strategies [80].

### Parkinson’s disease

Parkinson's disease (PD) is a degenerative neurological condition that gradually causes the death of dopaminergic neurons in the substantia nigra pars compacta (SNpc). This leads to motor symptoms, including tremors, stiffness, and slowness of movement [81]. Current treatments, including dopaminergic medications and deep brain stimulation, provide symptomatic relief but do not address the underlying neuronal loss or halt disease progression [82]. Stem cell-based therapies aim to replace lost dopaminergic neurons and restore motor function in PD [83]. It is important to note that Parkinson's disease is not simply characterized by the loss of dopaminergic neurons. The pathophysiology is complex, involving multiple neurotransmitter systems, protein aggregation, neuroinflammation, and dysfunction of various neural circuits. For stem cell therapies to be truly disease-modifying or curative, they would need to address these multiple aspects of PD pathology. Recent research has highlighted additional challenges, including the potential spread of alpha-synuclein pathology to transplanted cells and the need to restore broader neural circuit function beyond dopamine replacement [84].

#### Preclinical studies

Preclinical studies using animal models of PD have demonstrated the potential of various stem cell types, particularly ESCs and iPSCs, to differentiate into dopaminergic neurons and improve motor function. Transplantation of human ESC-derived dopaminergic neurons into the striatum of PD animal models has shown survival, integration, and functional recovery [85, 86]. Similarly, iPSC-derived dopaminergic neurons have demonstrated the ability to engraft, innervate the host striatum, and ameliorate motor deficits in PD models [87, 88]. However, challenges such as variability in differentiation efficiency, graft survival, and potential tumorigenicity need to be addressed [89]. PD models have also investigated MSCs for their neuroprotective and immunomodulatory properties [90, 91].

#### Clinical trials

Several clinical trials have investigated the safety and efficacy of stem cell therapies in PD patients. Early trials using fetal ventral mesencephalic (FVM) tissue grafts demonstrated variable outcomes, with some patients showing long-term clinical benefits and others developing graft-induced dyskinesias [92, 93]. More recently, clinical trials using human ESC-derived dopaminergic progenitors have shown promise. A phase 1/2 trial reported the safety and survival of transplanted cells in PD patients, with some evidence of motor improvement [94]. An ongoing phase 1 trial (NCT03119636) investigates the safety and efficacy of human ESC-derived dopaminergic progenitors in PD patients [95]. Clinical trials using autologous iPSC-derived dopaminergic neurons are also in the planning stages [96].

#### Challenges and future directions

While stem cell therapy for PD has made significant progress, several challenges remain. These include optimizing the differentiation and purification of dopaminergic neurons, ensuring graft survival and functional integration, and minimizing the risk of graft-induced dyskinesias [97]. Strategies to enhance graft survival, such as co-transplantation with supportive cell types or neuroprotective agents, are being explored [95]. The development of standardized protocols for cell manufacturing and quality control is also essential for the reproducibility and scalability of stem cell therapies [98]. Future directions in stem cell therapy for PD may involve gene editing technologies to correct disease-causing mutations in patient-specific iPSCs [99]. Cell encapsulation or bioengineered scaffolds may improve graft survival and integration [100]. Combinatorial approaches, such as the co-administration of neurotrophic factors or the use of neuroprotective agents, may enhance the therapeutic efficacy of stem cell therapies [101].

Stem cell therapy shows potential as a disease-modifying treatment for PD, aiming to replace lost dopaminergic neurons and restore motor function. Although there have been promising findings in preclinical studies and early clinical trials, additional research is required to tackle the obstacles and enhance the therapeutic approach. Continuing and upcoming clinical trials will offer valuable knowledge regarding the safety, effectiveness, and long-term advantages of stem cell therapies in PD.

### Multiple sclerosis

Multiple sclerosis (MS) is a long-lasting inflammatory condition of the CNS that involves the immune system attacking and damaging the protective covering of nerve fibers called myelin. This damage results in neurological problems and impairment [102]. Current therapies for MS primarily focus on immunomodulation and symptom management but do not effectively promote remyelination or prevent progressive neurodegeneration [103]. Stem cell-based therapies aim to promote remyelination, provide neuroprotection, and modulate the immune response in MS. Among neurological conditions, multiple sclerosis (MS) stands out as having the most advanced clinical applications of stem cell therapy. Autologous hematopoietic stem cell transplantation (aHSCT) is now routinely used in medical centers worldwide to treat aggressive forms of MS. This approach aims to 'reset' the immune system and halt disease progression [104].

#### Preclinical studies

Preclinical studies using animal models of MS, such as experimental autoimmune encephalomyelitis (EAE), have demonstrated the potential of various stem cell types to promote remyelination and ameliorate disease progression. Transplantation of NSCs or oligodendrocyte progenitor cells (OPCs) derived from ESCs or iPSCs has shown the ability to differentiate into mature oligodendrocytes, promote remyelination, and improve functional outcomes in EAE models [105, 106]. MSCs have also been extensively studied in MS models for their immunomodulatory and neuroprotective properties [107]. MSCs have been shown to reduce neuroinflammation, suppress autoreactive T cells, and promote the generation of regulatory T cells, leading to improved clinical outcomes in EAE [108, 109].

#### Clinical trials

Several clinical trials have investigated the safety and efficacy of stem cell therapies in MS patients. aHSCT has been explored as a potential treatment for aggressive forms of MS to reset the immune system and halt disease progression. Comparing aHSCT to disease-modifying therapies found that aHSCT was superior in preventing disease progression and achieving sustained improvement in neurological function. Long-term follow-up studies have shown that a significant proportion of patients remain free from disease activity for 5 years or more after treatment [110]. While some studies have shown promising results, with long-term stabilization or improvement of disability in a subset of patients, the procedure is associated with significant risks. It is currently reserved for select patients with highly active disease [111, 112]. Clinical trials using MSCs have also been conducted in MS patients, primarily focusing on safety and feasibility [113]. Intravenous administration of autologous MSCs is well-tolerated, with evidence of potential efficacy in reducing inflammatory activity and promoting neuroprotection [102, 114]. However, more extensive randomized controlled trials are needed to establish the long-term safety and efficacy of MSC-based therapies in MS.

#### Challenges and future directions

While stem cell therapies hold promise for the treatment of MS, several challenges need to be addressed. One of the main challenges is ensuring the survival, differentiation, and functional integration of transplanted cells in the host CNS [115]. Strategies to enhance graft survival and promote targeted migration to sites of demyelination are being explored [110]. Another challenge is the potential for graft rejection or the development of secondary autoimmunity [116]. The use of autologous or genetically modified stem cells and the development of improved immunosuppressive regimens may help mitigate these risks [117]. Future directions in stem cell therapy for MS may involve using gene editing technologies to create "off-the-shelf" cell products with enhanced remyelination capacity or immunomodulatory properties [118]. Developing biomaterials and tissue engineering approaches to create scaffolds that support cell survival and guide axonal regeneration is also an active area of research [119]. Combination therapies that target multiple aspects of MS pathology, such as neuroinflammation, oxidative stress, and mitochondrial dysfunction, may enhance the therapeutic potential of stem cell transplantation [120].

Stem cell-based therapies for MS have shown promising results in preclinical studies and early clinical trials. While challenges remain, advances in cell manufacturing, genetic engineering, and biomaterial science are expected to improve the safety, efficacy, and accessibility of stem cell therapies for MS in the future. Further research and well-designed clinical trials are needed to establish the optimal therapeutic approach and long-term benefits of stem cell transplantation in MS. The success of aHSCT in MS has led to its inclusion in treatment guidelines for highly active relapsing–remitting MS that is refractory to conventional therapies. However, patient selection is crucial, as the procedure carries risks and is most beneficial for younger patients with active inflammatory disease. Ongoing research is focused on optimizing aHSCT protocols, reducing treatment-related risks, and exploring its potential in progressive forms of MS. Additionally, other stem cell approaches, such as mesenchymal stem cell therapies, are being investigated for their potential neuroprotective and regenerative properties in MS [121].

### Stroke

Stroke is a leading cause of death and disability worldwide, characterized by the sudden loss of blood supply to the brain, resulting in neuronal damage and functional impairments [122]. Current treatments for stroke primarily focus on restoring blood flow and providing supportive care but do not effectively address the long-term neurological deficits [123]. Stem cell-based therapies promote neuronal repair, modulate inflammation, and enhance functional recovery in stroke [124].

#### Preclinical studies

Animal research investigating stroke has shown that different types of stem cells, such as MSCs, NSCs, and iPSCs, can enhance neuronal repair and functional outcomes. Studies have demonstrated that the transplantation of MSCs can decrease the extent of tissue damage caused by a lack of blood supply, regulate the inflammation of nerves, and improve the growth of new nerve cells and blood vessels in animal stroke models [125, 126]. NSCs, either from fetal tissue or generated from induced iPSCs, have shown the capacity to move towards the location of injury, transform into nerve cells and support cells, and enhance the restoration of function in stroke models [127, 128]. Nevertheless, the precise timing, method, and amount of stem cell administration and the sustained viability and incorporation of transplanted cells continue to be significant obstacles [129].

#### Clinical trials

Several clinical trials have investigated the safety and feasibility of stem cell therapies in stroke patients. A meta-analysis of early-phase clinical trials using MSCs in ischemic stroke patients reported a favorable safety profile and potential improvements in functional outcomes [130]. However, the efficacy of MSC transplantation in stroke remains to be established in larger, randomized controlled trials. The MASTERS trial, a phase 2 study of intravenous administration of bone marrow-derived MSCs in acute ischemic stroke patients, showed no significant improvement in functional outcomes at 90 days compared to placebo [128]. More recently, the TREASURE trial, a phase 2/3 study of intravenous administration of umbilical cord blood-derived MSCs in acute ischemic stroke patients, also failed to demonstrate a significant improvement in functional outcomes at 90 days [131]. These results highlight the need for further optimization of stem cell therapies for stroke, including the selection of patients most likely to benefit, the timing and route of administration, and the potential for combination therapies [127].

#### Challenges and future directions

While stem cell therapies hold promise for the treatment of stroke, several challenges need to be addressed. One of the main challenges is the limited survival and engraftment of transplanted cells in the ischemic brain [132]. Strategies to enhance cell survival, such as preconditioning or genetic modification of stem cells, are being explored [133]. Another challenge is the potential for off-target effects or the development of adverse events, such as tumorigenesis or stroke-associated infection [134]. The use of highly purified and well-characterized cell populations and rigorous safety monitoring will be essential for the clinical translation of stem cell therapies for stroke [135]. Future directions in stem cell therapy for stroke may involve using biomaterials and tissue engineering approaches to create a supportive microenvironment for transplanted cells and enhance their survival and differentiation [136]. Developing cell-free approaches, such as using extracellular vesicles or exosomes derived from stem cells, may also provide a more scalable and safe alternative to cell transplantation [137]. Combinatorial approaches, such as the co-administration of neuroprotective agents or the use of rehabilitation therapies, may enhance the therapeutic efficacy of stem cell transplantation [138].

While stem cell-based therapies for stroke have shown promising results in preclinical studies, the clinical translation of these approaches has been challenging. Further research is needed to optimize the therapeutic approach, including selecting the most appropriate stem cell type, the timing and route of delivery, and the potential for combination therapies. Well-designed clinical trials with larger sample sizes and more extended follow-up periods will be essential to establish the safety and efficacy of stem cell therapies for stroke.

### Amyotrophic lateral sclerosis

Muscle weakening, paralysis, and eventually death are the results of selective motor neuron loss in the brain and spinal cord that characterizes amyotrophic lateral sclerosis (ALS), a progressive neurodegenerative illness [139]. There are currently just a few ALS treatments available, and they mainly concentrate on supportive care and symptom control [140]. The goals of stem cell-based treatments for ALS are to reduce neuroinflammation, restore damaged motor neurons, and offer neuroprotection [141].

#### Preclinical studies

Preclinical studies using animal models of ALS, such as the SOD1 transgenic mouse model, have demonstrated the potential of various stem cell types to delay disease progression and extend survival. Transplantation of NSCs or motor neuron progenitors derived from ESCs or iPSCs has been shown to integrate into the spinal cord, form synaptic connections with host neurons, and improve motor function in ALS models [141, 142]. MSCs have also been extensively studied in ALS models for their immunomodulatory and neuroprotective properties [143]. Intrathecal or intravenous administration of MSCs has been shown to reduce neuroinflammation, protect against motor neuron loss, and prolong survival in ALS mice [144, 145]. However, the long-term survival and efficacy of transplanted cells in the diseased microenvironment of ALS remain significant challenges [146].

#### Clinical trials

Several early-phase clinical trials have investigated the safety and feasibility of stem cell therapies in ALS patients. Intraspinal transplantation of fetal spinal cord-derived NSCs in ALS patients is safe and well-tolerated, with some evidence of potential efficacy in slowing disease progression [147, 148]. However, a follow-up phase 2 trial did not significantly improve functional outcomes or survival compared to placebo [149]. Intrathecal administration of autologous MSCs has also been explored in ALS patients, focusing on safety and tolerability [137, 150]. While these early trials have provided proof-of-concept for the feasibility of stem cell transplantation in ALS, more extensive randomized controlled trials are needed to establish the efficacy of these approaches.

#### Challenges and future directions

Despite the promising preclinical results, the clinical translation of stem cell therapies for ALS faces several challenges. One of the main challenges is the complex and multifactorial nature of ALS pathogenesis, which may limit the therapeutic efficacy of cell replacement alone [151]. Strategies to enhance the survival, integration, and function of transplanted cells in the hostile microenvironment of ALS are being explored, such as the co-transplantation of supportive glial cells or the use of neuroprotective factors [152]. Another challenge is the potential for immune rejection or the development of adverse events, such as graft-induced dyskinesias or tumorigenesis [153]. The use of autologous or genetically modified stem cells and improved immunosuppressive regimens may help mitigate these risks [154].

Future directions in stem cell therapy for ALS may involve gene editing technologies to correct ALS-causing mutations in patient-specific iPSCs, which could then be differentiated into healthy motor neurons for transplantation [155]. Using biomaterials and tissue engineering approaches to create scaffolds that support cell survival and guide axonal regeneration is also an active area of research [156]. Combinatorial approaches, such as the co-administration of neuroprotective agents or the use of anti-inflammatory drugs, may enhance the therapeutic potential of stem cell transplantation [157]. Developing novel delivery methods, such as intramuscular or intravascular administration of stem cells, may provide a less invasive and more scalable approach for cell therapy in ALS [158].

Although preclinical research on stem cell-based therapy for ALS has yielded encouraging findings, the practical application of these strategies has been complex. More studies are required on the most suitable stem cell type, administration timing and route, and the possibility of combination therapies to optimize the therapeutic strategy. More significant sample numbers and extended follow-up periods in carefully planned clinical trials will be necessary to confirm the safety and effectiveness of stem cell treatments for ALS. Additionally, the development of successful stem cell-based treatments for this debilitating illness will depend on a deeper comprehension of the underlying mechanisms of ALS pathogenesis and the interactions between transplanted cells and the host milieu.

### Huntington’s disease

Huntington's disease (HD) is an inherited neurodegenerative disorder caused by a trinucleotide repeat expansion in the huntingtin gene, leading to the production of a mutant huntingtin protein that causes progressive neuronal loss and dysfunction, particularly in the striatum and cortex [159]. Current treatments for HD are limited and primarily focus on managing symptoms, such as chorea and psychiatric disturbances [160]. Stem cell-based therapies aim to replace lost neurons, provide neuroprotection, and modulate neuroinflammation in HD [161].

#### Preclinical studies

Preclinical studies using animal models of HD, such as the R6/2 and YAC128 transgenic mouse models, have demonstrated the potential of various stem cell types to improve motor function, reduce neuronal loss, and extend survival. Transplantation of fetal striatal tissue or NSCs derived from ESCs or iPSCs has been shown to integrate into the striatum, form synaptic connections with host neurons, and ameliorate motor deficits in HD mice [162, 163]. MSCs have also been explored in HD models for their immunomodulatory and neuroprotective properties [164]. Intrastriatal or intravenous administration of MSCs has been shown to reduce neuroinflammation, increase neurotrophic factor levels, and improve motor function in HD mice [165, 166]. However, the long-term survival and efficacy of transplanted cells in the diseased microenvironment of HD remain significant challenges [167].

#### Clinical trials

To date, few clinical trials have investigated the safety and feasibility of stem cell therapies in HD patients. A phase 1 trial of fetal striatal tissue transplantation in HD patients demonstrated the safety and feasibility of the approach, with some evidence of graft survival and clinical benefit [168]. However, a follow-up study found that the transplanted cells developed HD-like pathology over time, suggesting that cell replacement alone may not halt disease progression [169]. Recently, a phase 1/2 trial of intrastriatal transplantation of human ESC-derived neural progenitors in HD patients has been initiated (NCT03252080) [170]. This trial aims to assess the approach's safety, tolerability, and preliminary efficacy, with results expected in the coming years.

#### Challenges and future directions

While stem cell-based therapies for HD hold promise, several challenges must be addressed for successful clinical translation. One of the main challenges is the potential for transplanted cells to acquire HD-related pathology over time due to the presence of the mutant huntingtin protein in the host environment [171]. Strategies to mitigate this risk, such as genetically corrected autologous iPSCs or the co-transplantation of neuroprotective factors, are being explored [172]. Another challenge is the need for targeted cell delivery to the affected brain regions, as widespread neuronal loss and circuit dysfunction occur in HD [173]. Developing advanced imaging techniques and stereotactic surgery methods may help guide precise cell transplantation [154]. Future directions in stem cell therapy for HD may involve gene editing technologies, such as CRISPR–Cas9, to correct the HTT mutation in patient-specific iPSCs, which could then be differentiated into healthy striatal neurons for transplantation [155]. Using biomaterials and tissue engineering approaches to create scaffolds that support cell survival and guide axonal regeneration is also an active area of research [156]. Combinatorial approaches, such as the co-administration of neuroprotective agents or the use of anti-inflammatory drugs, may enhance the therapeutic potential of stem cell transplantation [174]. Developing novel delivery methods, such as intracerebroventricular or intrathecal administration of stem cells, may provide a less invasive and more widespread approach for cell therapy in HD [175].

In summary, whereas preclinical research on stem cell-based treatments for Huntington's disease has yielded encouraging outcomes, the clinical application of these strategies is still in its infancy. More studies are required on the most suitable stem cell type, administration timing and route, and the possibility of combination therapies to optimize the therapeutic strategy. To prove that stem cell therapies for HD are safe and effective, well-designed clinical trials with bigger sample sizes and longer follow-up times will be necessary. Additionally, the development of successful stem cell-based treatments for this debilitating illness will depend on a deeper comprehension of the molecular mechanisms driving HD pathogenesis and the interactions between transplanted cells and the host milieu.

### Spinal cord injury

A severe disorder known as spinal cord injury (SCI) causes the loss of motor and sensory function below the site of the damage, which frequently leads to permanent paralysis and impairment [176]. The main goals of current SCI treatments are to stabilize the spine, stop more injury, and encourage recovery [177]. The goals of stem cell-based treatments for spinal cord injury (SCI) include glia and missing neuron replacement, axonal regeneration, and inflammation response modulation [178].

#### Preclinical studies

The potential of different types of stem cells to support functional recovery and regeneration has been proven in preclinical research utilizing animal models of spinal cord injury. It has been demonstrated that transplanting NSCs or neural progenitor cells (NPCs) derived from ESCs or iPSCs into the injured spinal cord can improve motor function in rodent and primate models of SCI by promoting the differentiation of neurons and glia and forming synaptic connections with host neurons. Since MSCs exhibit immunomodulatory, neuroprotective, and pro-angiogenic qualities, they have also been the subject of substantial research in SCI models [179]. It has been demonstrated that administering MSCs intravenously or intraspinally to SCI mice can decrease inflammation, encourage tissue sparing, and improve functional recovery [180, 181]. Nonetheless, there are still significant obstacles to overcome, including the best time, method, and dosage for delivering stem cells and the integration and long-term survival of transplanted cells [182].

#### Clinical trials

Several clinical trials have investigated the safety and feasibility of stem cell therapies in SCI patients. A systematic review and meta-analysis of clinical trials using MSCs in SCI patients found no serious adverse events related to cell transplantation and some evidence of functional improvement [183]. However, the included studies were small, heterogeneous, and lacked appropriate controls, highlighting the need for more extensive, well-designed trials to establish the efficacy of MSC therapy in SCI [184]. A phase 2 trial of intramedullary transplantation of human ESC-derived oligodendrocyte progenitor cells in subacute SCI patients (NCT02302157) has recently been completed, with results pending publication [185]. Other ongoing or planned trials investigate the safety and efficacy of various stem cell types in SCI patients, including NSCs, NPCs, and autologous bone marrow-derived MSCs [186].

#### Challenges and future directions

While stem cell-based therapies for SCI hold promise, several challenges must be addressed for successful clinical translation. One of the main challenges is the complex and dynamic nature of the injury microenvironment, which may limit the survival, differentiation, and integration of transplanted cells [187]. Strategies to enhance cell survival and promote targeted differentiation, such as co-delivering neuroprotective factors or biomaterials and tissue engineering approaches, are being explored [188, 189]. Another challenge is the potential for adverse events, such as neuropathic pain, autonomic dysreflexia, or tumor formation, following stem cell transplantation [190]. Careful patient selection, rigorous safety monitoring, and long-term follow-up will be essential to mitigate these risks [184].

Future directions in stem cell therapy for SCI may involve gene editing technologies to engineer stem cells with enhanced regenerative properties, such as increased neurotrophic factor secretion or improved myelination capacity [191]. The development of advanced biomaterials and tissue engineering approaches to create scaffolds that mimic the natural extracellular matrix and guide axonal regeneration is also an active area of research [154]. Combinatorial approaches, such as the co-administration of rehabilitation therapy or the use of electrical stimulation, may enhance the therapeutic potential of stem cell transplantation [192]. Additionally, identifying reliable biomarkers and imaging techniques to monitor the survival, differentiation, and integration of transplanted cells in vivo will be critical for optimizing and individualizing stem cell therapies for SCI [193].

Stem cell-based therapies for SCI have shown promising results in preclinical studies, with growing evidence of safety and feasibility in early clinical trials. However, further research is needed to optimize the therapeutic approach, including selecting the most appropriate stem cell type, the timing and route of delivery, and the potential for combination therapies. Well-designed, randomized controlled trials with larger sample sizes, longer follow-up periods, and standardized outcome measures will be essential to establish the efficacy of stem cell therapies for SCI. Additionally, a deeper understanding of the molecular mechanisms underlying SCI pathophysiology and the interactions between transplanted cells and the host microenvironment will be critical for developing safe and effective stem cell-based therapies for this devastating condition.

### Traumatic brain injury

Traumatic brain injury (TBI) is a leading cause of death and disability worldwide, resulting from sudden physical damage to the brain due to external forces, such as falls, vehicle accidents, or violence [194]. The primary injury initiates a cascade of secondary injury mechanisms, including neuroinflammation, oxidative stress, excitotoxicity, and apoptosis, leading to progressive neuronal loss and dysfunction [195]. Current treatments for TBI primarily focus on minimizing secondary injury, managing intracranial pressure, and providing rehabilitation to promote functional recovery [196]. However, there are no effective therapies to reverse the damage and restore function in chronic TBI patients [197]. Stem cell-based therapies aim to replace lost neurons and glia, modulate the inflammatory response, and promote neurogenesis and angiogenesis in TBI [198].

#### Preclinical studies

Preclinical studies using animal models of TBI have demonstrated the potential of various stem cell types, including neural stem/progenitor cells (NSPCs), MSCs, and HSCs, to promote functional recovery after TBI. Transplantation of NSPCs derived from ESCs or iPSCs into the injured brain has been shown to differentiate into neurons and glia, form synaptic connections with host neurons, and improve cognitive and motor function in rodent models of TBI [199, 200]. MSCs have also been extensively studied in TBI models for their immunomodulatory, neuroprotective, and pro-angiogenic properties [201]. Intravenous or intracerebral administration of MSCs has been shown to reduce inflammation, promote neurogenesis and angiogenesis, and enhance functional recovery in TBI animals [202, 203]. HSCs mobilized from the bone marrow have been shown to migrate to the injured brain, differentiate into microglia and neurons, and improve cognitive function in rodent models of TBI [204]. While preclinical studies of stem cell therapies for TBI have shown promise, significant challenges remain in translating these approaches to clinical practice. Further research is needed to optimize cell types, delivery methods, and timing of intervention. Importantly, the complex and heterogeneous nature of TBI may require combinatorial approaches rather than relying solely on cell replacement strategies.

#### Clinical trials

Several early-phase clinical trials have investigated the safety and feasibility of stem cell therapies in TBI patients. A phase 1/2a study of intravenous administration of autologous bone marrow-derived mononuclear cells (BMMNCs) in acute severe TBI patients demonstrated safety and a trend towards improved neurological outcomes [205]. Another phase 1 study of intracerebral transplantation of human NSCs in chronic TBI patients showed safety and feasibility, with some evidence of improved neurological function [206]. However, more extensive randomized controlled trials are needed to establish the efficacy of these approaches in improving functional outcomes and quality of life in TBI patients [207].

#### Challenges and future directions

While stem cell-based therapies for TBI hold promise, several challenges must be addressed for successful clinical translation. One of the main challenges is the heterogeneity of TBI, which can vary in terms of the mechanism, location, and severity of injury, as well as the age and comorbidities of the patient [208]. Developing personalized stem cell therapies tailored to each patient's specific needs may be necessary to maximize therapeutic efficacy [209]. Another challenge is the potential for adverse events, such as seizures, infection, or tumorigenesis, following stem cell transplantation [190]. Careful patient selection, rigorous safety monitoring, and long-term follow-up will be essential to mitigate these risks [210].

Future directions in stem cell therapy for TBI may involve gene editing technologies to enhance the regenerative properties of transplanted cells, such as overexpressing neurotrophic factors or anti-inflammatory cytokines [211]. Research is also being done using biomaterials and tissue engineering techniques to make scaffolds that resemble the extracellular matrix seen in nature and offer a favorable environment for cell survival and development [212]. The therapeutic potential of stem cell transplantation may be increased by combinatorial techniques, including co-administration of neuroprotective drugs, neurorestorative treatments, or rehabilitation [213]. Furthermore, developing noninvasive imaging modalities like PET and MRI to track the migration, survival, and differentiation of transplanted cells in vivo would be essential for customizing and streamlining stem cell treatments for traumatic brain injury [214].

Stem cell-based therapies for TBI have shown promising results in preclinical studies, with early evidence of safety and feasibility in clinical trials. However, further research is needed to optimize the therapeutic approach, including selecting the most appropriate stem cell type, the timing and route of delivery, and the potential for combination therapies. Well-designed, randomized controlled trials with larger sample sizes, longer follow-up periods, and standardized outcome measures will be essential to establish the efficacy of stem cell therapies for TBI. Additionally, a deeper understanding of the complex pathophysiology of TBI and the mechanisms underlying the therapeutic effects of stem cells will be critical for developing safe and effective regenerative medicine approaches for this devastating condition.

### Epilepsy

Repeated, unprovoked seizures are a hallmark of epilepsy, a chronic neurological illness caused by abnormally high levels of aberrant brain neuronal activity [215]. Even though antiepileptic medications (AEDs) are the cornerstone of epilepsy treatment, over one-third of patients still do not respond to medication [216]. Intending to reestablish the proper balance between excitement and inhibition in the epileptic brain, stem cell-based therapies have become a viable adjunctive or alternative therapeutic option for drug-resistant epilepsy [217].

#### Preclinical studies

Preclinical studies using animal models of epilepsy have demonstrated the potential of various stem cell types, particularly GABAergic interneuron progenitors and MSCs, to suppress seizures and modify the underlying disease pathology. Transplantation of GABAergic interneuron progenitors derived from ESCs or iPSCs into the hippocampus or other seizure-prone regions has been shown to engraft, differentiate into functional GABAergic interneurons, and reduce seizure frequency and severity in rodent models of epilepsy [218, 219]. These effects are mediated by the synaptic integration of the transplanted cells into the host circuitry and the enhancement of inhibitory neurotransmission [220]. MSCs have also shown promise in preclinical epilepsy models, exerting anticonvulsant and neuroprotective effects through the secretion of neurotrophic factors and the modulation of inflammatory responses [221, 222]. However, the long-term efficacy and safety of stem cell therapies in epilepsy remain to be established [223].

#### Clinical trials

To date, few clinical trials have investigated the safety and efficacy of stem cell therapies in epilepsy patients. A phase 1 trial of autologous bone marrow-derived mononuclear cells (BMMNCs) administered intravenously in children with refractory epilepsy demonstrated safety and feasibility, with some evidence of reduced seizure frequency [224]. Another pilot study of intracerebral transplantation of autologous BMMNCs in adult patients with drug-resistant mesial temporal lobe epilepsy also showed safety and potential efficacy in reducing seizure frequency [225]. However, these early-stage trials are limited by small sample sizes, lack of control groups, and short follow-up periods, highlighting the need for larger, well-designed, randomized controlled trials to establish the efficacy of stem cell therapies in epilepsy [226].

#### Challenges and future directions

While stem cell-based therapies for epilepsy hold promise, several challenges must be addressed for successful clinical translation. One of the main challenges is the complex and multifactorial nature of epilepsy, which may require tailored stem cell therapies targeting specific epileptogenic mechanisms in each patient [227]. Another challenge is the potential for adverse events, such as graft rejection, tumor formation, or worsening of seizures, following stem cell transplantation [228]. Careful patient selection, rigorous safety monitoring, and long-term follow-up will be essential to mitigate these risks [210].

Future directions in stem cell therapy for epilepsy may involve gene editing technologies to create stem cell-derived GABAergic interneurons with enhanced anticonvulsant properties or reduced immunogenicity [229]. The development of advanced delivery methods, such as stereotactic surgery or convection-enhanced delivery, to achieve targeted and controlled transplantation of stem cells into the epileptic focus is also an active area of research [230]. Combinatorial approaches, such as the co-administration of neuroprotective agents or the use of gene therapy to overexpress seizure-suppressing molecules, may enhance the therapeutic potential of stem cell transplantation [231]. Additionally, identifying reliable biomarkers and advanced neuroimaging techniques to guide patient selection, monitor the fate of transplanted cells, and assess the efficacy of stem cell therapies in vivo will be critical for optimizing and individualizing stem cell-based treatments for epilepsy [232].

Stem cell-based therapies for epilepsy have shown promise in preclinical studies, with some evidence of safety and feasibility in early clinical trials. However, further research is needed to optimize the therapeutic approach, including selecting the most appropriate stem cell type, the timing and route of delivery, and the potential for combination therapies. Well-designed, randomized controlled trials with larger sample sizes, longer follow-up periods, and standardized outcome measures will be essential to establish the efficacy of stem cell therapies for epilepsy. Additionally, a deeper understanding of the complex pathophysiology of epilepsy and the mechanisms underlying the therapeutic effects of stem cells will be critical for developing safe and effective regenerative medicine approaches for this challenging neurological disorder.

### Other emerging applications

In addition to the neurological disorders discussed above, stem cell-based therapies have shown potential for the treatment of various other neurological conditions, such as cerebral palsy, autism spectrum disorder (ASD), and peripheral nerve injuries.

#### Cerebral palsy

A set of lifelong mobility abnormalities known as cerebral palsy are brought on by harm to the developing brain and first manifest in early childhood [233]. Current treatments for cerebral palsy primarily focus on managing symptoms and improving function through physical therapy, occupational therapy, and medications [234]. Stem cell-based therapies, particularly umbilical cord blood (UCB) cells and MSCs, have shown promise in preclinical and early clinical studies for cerebral palsy [235]. These cells have been shown to exert neuroprotective, anti-inflammatory, and pro-angiogenic effects, promoting brain repair and functional recovery [236, 237]. However, more extensive randomized controlled trials are needed to establish stem cell therapies' efficacy and long-term safety for cerebral palsy [238].

#### Autism spectrum disorder

Autism spectrum disorder (ASD) is a neurodevelopmental disorder characterized by deficits in social communication and interaction, along with restricted and repetitive patterns of behavior [239]. While behavioral and educational interventions are the mainstay of treatment for ASD, there are no effective pharmacological therapies to address the core symptoms [240]. Stem cell-based therapies, particularly MSCs and NSCs, have shown potential in preclinical studies to modulate the immune system, promote synaptic plasticity, and improve behavioral outcomes in animal models of ASD [241, 242]. A few small clinical studies have investigated the safety and feasibility of stem cell therapies in ASD patients, with some evidence of improved behavioral and cognitive function [243, 244]. However, these studies are limited by small sample sizes, lack of control groups, and short follow-up periods, highlighting the need for larger, well-designed clinical trials to establish the efficacy and safety of stem cell therapies for ASD [245].

#### Peripheral nerve injuries

Peripheral nerve injuries caused by trauma, surgery, or disease can lead to sensory and motor deficits, neuropathic pain, and reduced quality of life [246]. Current treatments for peripheral nerve injuries primarily focus on surgical repair, physical therapy, and pain management [246]. Stem cell-based therapies, particularly Schwann cells, MSCs, and adipose-derived stem cells (ADSCs), have shown promise in preclinical studies to promote nerve regeneration, remyelination, and functional recovery [247–249]. These cells can be transplanted directly into the injured nerve or delivered through nerve guidance conduits or other biomaterial scaffolds [250]. A few early-stage clinical trials have investigated the safety and feasibility of stem cell therapies for peripheral nerve injuries, with some evidence of improved sensory and motor function [176, 251]. However, further research is needed to optimize the therapeutic approach and establish stem cell therapies' long-term efficacy and safety for peripheral nerve injuries [247].

### Challenges and future directions

While stem cell-based therapies for these neurological disorders hold promise, several challenges must be addressed for successful clinical translation. These include the heterogeneity of the patient population, the complex and multifactorial nature of the underlying pathology, and the potential for adverse events following stem cell transplantation [252]. Future directions may involve the development of personalized stem cell therapies tailored to each patient's specific needs, the use of gene editing technologies to enhance the therapeutic properties of stem cells, and the exploration of combinatorial approaches to enhance the efficacy of stem cell transplantation [253–255].

Stem cell-based therapies have shown potential for treating neurological disorders beyond the well-studied conditions discussed earlier. However, further preclinical and clinical research is needed to establish these approaches' safety, efficacy, and long-term benefits. As regenerative medicine advances, it is hoped that stem cell-based therapies will become a viable treatment option for a wide range of neurological disorders, improving the quality of life for patients and their families.

## Conclusion and future perspectives

Stem cell-based therapies for neurological disorders have made significant progress in recent years, with promising results from preclinical studies and early clinical trials. As highlighted in this comprehensive review, various stem cell types, including NSCs, MSCs, and iPSCs, have shown potential for the treatment of a wide range of neurological conditions, such as Parkinson's disease, Alzheimer's disease, multiple sclerosis, stroke, spinal cord injury, and traumatic brain injury.

The therapeutic potential of stem cells in neurological disorders is primarily attributed to their ability to replace lost or damaged neural cells, modulate the immune system, promote endogenous repair mechanisms, and provide trophic support to the injured or diseased nervous system. However, the exact mechanisms underlying the therapeutic effects of stem cells are not fully understood. They may vary depending on the specific neurological condition and the type of stem cells used.

Despite the encouraging progress, several challenges need to be addressed to realize the full potential of stem cell-based therapies for neurological disorders. These include optimizing stem cell sources, differentiation protocols, and delivery methods to ensure the therapeutic approach's safety, efficacy, and reproducibility. The potential for tumorigenicity, immune rejection, and other adverse events following stem cell transplantation also requires careful consideration and long-term monitoring. Furthermore, the complex and multifactorial nature of many neurological disorders may require combinatorial approaches that target multiple pathogenic mechanisms, such as gene therapy, neuroprotective agents, or rehabilitation in conjunction with stem cell transplantation.

Several critical research areas must be prioritized to address these challenges and accelerate the clinical translation of stem cell-based therapies for neurological disorders. These include the development of standardized protocols for the generation, characterization, and banking of clinical-grade stem cells, the establishment of robust preclinical models that more accurately recapitulate human neurological disorders, and the design of well-controlled clinical trials with appropriate patient populations, outcome measures, and follow-up periods.

In addition to technological advancements, the successful clinical translation of stem cell-based therapies for neurological disorders will require a collaborative and multidisciplinary approach involving basic scientists, clinicians, industry partners, regulatory agencies, and patient advocates. Ethical, legal, and social implications of stem cell research and therapy must also be carefully addressed through ongoing dialogue and public engagement.

Looking to the future, the field of stem cell-based therapies for neurological disorders is poised for exciting developments and breakthroughs. The convergence of stem cell biology with other cutting-edge technologies, such as gene editing, single-cell genomics, organoid models, and advanced neuroimaging, holds great promise for developing personalized and targeted therapies for neurological disorders. The increasing understanding of the complex interplay between the nervous system, immune system, and microbiome may also open up new avenues for stem cell-based therapies that harness the body's intrinsic regenerative capacity.

In conclusion, while stem cell-based therapies for neurological disorders are still in their early stages of development, the progress made so far is encouraging and holds great promise for the future. With continued research, collaboration, and innovation, it is hoped that stem cell-based therapies will become a safe, effective, and accessible treatment option for millions worldwide affected by neurological disorders, improving their quality of life and reducing the burden on healthcare systems. As the field continues to evolve, it will be essential to maintain a balanced and evidence-based perspective, acknowledging both the potential and the limitations of stem cell-based therapies and to ensure that the interests of patients and the public are always at the forefront of scientific endeavors.

## Data Availability

No datasets were generated or analysed during the current study.
